# Hypoxia Up-Regulates Galectin-3 in Mammary Tumor Progression and Metastasis

**DOI:** 10.1371/journal.pone.0134458

**Published:** 2015-07-29

**Authors:** Joana T. de Oliveira, Cláudia Ribeiro, Rita Barros, Catarina Gomes, Augusto J. de Matos, Celso A. Reis, Gerard R. Rutteman, Fátima Gärtner

**Affiliations:** 1 Instituto de Investigação e Inovação em Saúde, Universidade do Porto, Porto, Portugal; 2 Institute of Molecular Pathology and Immunology (IPATIMUP), University of Porto, Porto, Portugal; 3 Instituto de Ciências Biomédicas de Abel Salazar (ICBAS), University of Porto, Porto, Portugal; 4 Faculty of Veterinary Medicine of the Lusophone University of Humanities and Technologies, Lisbon, Portugal; 5 Animal Science and Study Central (CECA), Food and Agrarian Sciences and Technologies Institute (ICETA), University of Porto, Porto, Portugal; 6 Department of Clinical Sciences of Companion Animals, Faculty of Veterinary Medicine, Utrecht University, Utrecht, The Netherlands; 7 Specialist Veterinary Centre De Wagenrenk, Utrecht, The Netherlands; University of Tennessee Health Science Center, UNITED STATES

## Abstract

The tumor microenvironment encompasses several stressful conditions for cancer cells such as hypoxia, oxidative stress and pH alterations. Galectin-3, a well-studied member of the beta-galactoside-binding animal family of lectins has been implicated in multiple steps of metastasis as cell-cell and cell-ECM adhesion, promotion of angiogenesis, cell proliferation and resistance to apoptosis. However, both its aberrantly up- and down-regulated expression was observed in several types of cancer. Thus, the mechanisms that regulate galectin-3 expression in neoplastic settings are not clear. In order to demonstrate the putative role of hypoxia in regulating galectin-3 expression in canine mammary tumors (CMT), *in vitro* and *in vivo* studies were performed. In malignant CMT cells, hypoxia was observed to induce expression of galectin-3, a phenomenon that was almost completely prevented by catalase treatment of CMT-U27 cells. Increased galectin-3 expression was confirmed at the mRNA level. Under hypoxic conditions the expression of galectin-3 shifts from a predominant nuclear location to cytoplasmic and membrane expressions. In *in vivo* studies, galectin-3 was overexpressed in hypoxic areas of primary tumors and well-established metastases. Tumor hypoxia thus up-regulates the expression of galectin-3, which may in turn increase tumor aggressiveness.

## Introduction

Galectin-3 is a unique member of the family of galectins. It is a carbohydrate-binding protein which mediates cell—cell and cell—extracellular matrix (ECM) interactions and that has been implicated in several key steps of the cancer metastatic process [1, 2] and drug resistance [3, 4]. The relationship between the expression of galectin-3 and cancer behavior is controversial and the mechanisms controlling its expression remain unclear [5, 6].

Our previous studies demonstrated that the expression of galectin-3 and galectin-3-binding sites is dynamic and seems to be, at least in part, microenvironment-related [7]. Altered glycan-galectin dynamics is likely to facilitate the detachment of tumor cells from primary sites and thus increase their invasive and metastatic capabilities [8–11]. Galectin-3 was shown to be down-regulated in primary canine mammary carcinomas when compared to adenomas [12] suggesting a possible selective advantage for malignant growth when the level of this lectin is decreased [13, 14]. Despite the low expression of galectin-3 in most malignant tumor areas [12, 15–17] tumor cells surrounding necrotic areas are found to express more galectin-3. This suggests that a hypoxic microenvironment might increase its expression [12, 18–20], which in turn might be related to increased aggressiveness of tumor cells [21]. Galectin-3 has also been found to act as a chemo-attractant to endothelial cells and to stimulate neovascularization through vascular endothelial growth factor (VEGF) in the tumor stroma [22], thus contributing to the establishment of an escape route for metastatic cells [12, 22]. Furthermore, galectin-3 confers resistance to anoikis [23] to these metastatic cells, hence contributing to their survival in the blood flow, a crucial rate-limiting step of metastasis [12].

Tumor hypoxic regions are those in which cells suffer not only from lack of oxygen but also from glucose and amino acids deprivation, high lactate concentration and oxidative stress. In solid tumors, hypoxia is primarily a pathophysiological consequence of the high tumor growth with lagging angiogenesis, and is one of the major stress sources for both cancer and normal cells [18, 24, 25]. The cell response to hypoxia is mainly mediated by HIF-1α. [18, 26]. It has been demonstrated that hypoxia-related changes are associated to poor prognosis and to increased chemo and radiotherapy resistance [18]. HIF-1α up-regulates several genes in order to promote survival under hypoxic conditions, such is the case of the glucose transporter GLUT-1 and the proangiogenic VEGF [27, 28]. HIF-1α has also been shown to up-regulate the expression of galectin-3 in a non-neoplastic context, that of the hypoxic nucleus pulposus of the intervertebral disc [20].

In accordance with the above, in breast cancer, the presence of tumor necrosis has been associated to a decreased survival rate [29, 30]. Recently, we and Chammas’s group showed galectin-3 overexpression around necrotic areas of canine mammary tumors (CMT) and breast ductal carcinoma *in situ*, respectively [12, 31] and suggested a link between hypoxia and the expression of different glycoconjugates *in vivo*. This may imply that galectin-3 expression is regulated both at the transcriptional and post-transcriptional levels [7, 32]. Little is known about the mechanisms underlying these phenomena and the direct role of hypoxia remains unknown.

In this study we report that galectin-3 expression is associated with tumor microenvironment hypoxia in canine mammary cancer cells. Interestingly, hypoxia also leads to a shift in galectin-3 subcellular localization, and its effects are precluded by detoxification. Based on these results, we propose a model to reconcile the overexpression of galectin-3 in necrosis surrounding areas of canine mammary cancer lesions and suggest that this might be a critical player in metastasis.

## Material and Methods

### Cell lines and culture conditions

In this study, we used a highly metastatic CMT cell line (CMT-U27) provided by Professor Eva Hellmén from Sweden)[33]. Cells were cultured at 37°C in a humidified 5% CO_2_ incubator (Heraeus, Thermo Electron Corporation) and maintained in RPMI 1640 medium supplemented with 10% of fetal bovine serum and 1% of penicillin and streptomycin.

#### Hypoxia Assay

For hypoxic stimulation, the same CMT-U27 cell line was cultured for 6, 12 and 24 hours in a modulator incubator (Binder) with a gas mixture of 0.1% O_2_, 5% CO_2_ with the balance of nitrogen at 37°C and maintained in RPMI 1640 medium supplemented with 10% of fetal bovine serum (FBS) and 1% penicillin and streptomycin.

#### H2O2 Assay

A 34,5% W/V H_2_O_2_ stock solution (Merck, Germany) was dissolved with RPMI 1640 to make a 25mM H_2_O_2_ solution. Fifty (50) μM of H_2_O_2_ were prepared in the culture medium, and cells were placed in culture for 12 hours.

#### Catalase treatment

Cultured cells were incubated with RPMI 1640 supplemented with 4μM of catalase (SIGMA, USA) for 12 hours.

### Double-labeling Immunofluorescence in cells

For immunofluorescence visualization of galectin-3 and Glucose Transporter-1 (GLUT-1) in hypoxic conditions, cells were grown for 12 hours on cover glasses pre-coated for 3 hours with RPMI 1640 medium supplemented with 10% fetal bovine serum in a modulator incubator with a gas mixture of 0.1% O_2_, 5%CO_2_ and the balance of nitrogen at 37°C. The cells were then fixed for 10 minutes in ice-cold methanol and blocked with non-immune serum diluted in 10% BSA for 30 minutes. After that, monoclonal rat anti-galectin-3 antibody (Bioscience) (1:200) and polyclonal rabbit anti- GLUT-1 (Abcam) (1:50) were diluted in 5% bovine serum albumin (BSA), and incubated overnight. After three washes with PBS, cells were incubated with Alexa 588-conjugated with donkey anti-rat antibody (Invitrogen) and Alexa 594-conjugated with goat anti-rabbit antibody (1:200) (Invitrogen) respectively, diluted in 5% BSA for 30 minutes in the dark at room temperature, followed by incubation with DAPI (1:100) in PBS and then mounted in vector shield (Vector). Cells were analyzed with Axio Imager Z1 (Zeiss) Fluorescence microscope and TCS SP5 II (Leica) Laser Scanning Confocal.

### Flow Cytometry

CMT-U27 cell line was plated in 6-well plates, 7,5x105 cells per well, and grown at 37°C for 12 hours with 21% of O_2_ (normoxic conditions) or 0.1% of O_2_ (hypoxic conditions) in medium containing 10% of FBS. Then cells were harvested, washed with wash solution (PBS containing 1% of BSA) and fixed in methanol for 10 minutes. Once washed, cells were incubated with anti-galectin-3 (M3/38) for 1 hour at room temperature. After 3 washes with PBS contained 1% of BSA, cells were incubated with alexa 488-conjugated donkey anti-rat secondary antibody for 30 minutes. Nuclei were stained with DAPI for 15 minutes. Then, cells were analyzed using a FACS Canto v.2 flow cytometer. The experiments were performed three independent times.

### Protein extraction and Western Blot

Cultured cells were washed twice in cold PBS, then total proteins were scrapped with RIPA (20mM Tris, pH7.2, 10mM EDTA, 0.3 M NaCL, 0,1% Triton X-100, 0,005%tween -20) containing protease inhibitors (1 mM phenyl methyl sulfonyl fluoride (PMSF), 1 mM sodium orthovanadate (Na_3_VO_4_) and complete protease inhibitor cocktail (Roche Applied Science) and incubated on ice for 15 minutes. The lysates were then centrifuged, the supernatants collected, and protein content was quantified by BCA Protein Assay Reagent (Thermo Scientific), according to the manufacturer’s instructions. Proteins were run on 12% SDS-poly acrylamide gels and transferred by electro blotting to nitrocellulose membranes (Amersham, Biosciences). The membranes were blocked with 5% non-fat dry milk in TBS-T (50mM Tris, pH 7.6, 150mM NaCl, 0,005%Tween 20) and were incubated overnight at 4°C in 2% nonfat dry milk in TBS-T with anti-galectin-3 (1:400, Bioscience); anti- GLUT-1 (1:400, Abcam), anti-HIF1α (1:500, BD transduction laboratories) and anti-actin (1:2000, Santa Cruz Biotechnology). The primary antibodies were revealed using the appropriate peroxidase-conjugated secondary antibodies (1:2000). Immunolabeling was detected using the enhanced chemiluminescence reagent (ECL; Amersham Biosciences). Actin levels were used to normalize protein amount, and quantification of western blots was performed using Gs-800 calibrated dimensitometer (Bio Rad). These experiments were repeated 3 times.

### RNA extraction and Real-Time polymerase chain reaction

At the end of each treatment period, total RNA was extracted from cells using TRI Reagent (Sigma) according to the manufacturer's instructions. Real Time-PCR was used to determine *galectin-3*, *HIF1-α*, *GLUT-1* and *GAPDH* expression. Briefly, 3 μg of RNA were primed with random hexamers and reversely transcribed using Superscript II Reverse Transcriptase kit (Invitrogen) in a final volume of 20 μL. Two microliters of a 1:10 dilution of this mixture were amplified with SYBR Green (Applied Bio systems) and primers at a final concentration of 300 nM each. Each sample was amplified in triplicate and specificity confirmed by dissociation analysis. Analysis of mRNA expression was performed in a fluorescence reader ABI Prism 7500 Fast. The level of 18S RNA in each sample was measured and used for normalization of target gene abundance. Primer sequences are listed below.

GAL-3: (5′CAGGCAGCTTTTCCATTCGA3′/5′ACTGCAACAAATGGGCATCA3′);

HIF-1α: (5’GCGTGTAAGGAAGCTTCTGG3’/5’GGTTCTCACAGATGATGGTG3’)

GLUT-1: (5’CAACCGCAATGAGGAGAACC3’/5’GGAGAAGAAGGTCACCATCC3’);

GAPDH: (5’ACGGTCAAGGCTGAGAATGG3’/5’CTCAGCACCAGCATCACCC3’)

These experiments were repeated twice.

### *In situ* RNA labelling

Canine galectin-3 RNA was detected *in situ* using a set of Stellaris RNA fluorescence *in situ* hybridization (FISH) probes (Biosearch Technologies, Novato, CA, USA). The 39 probes were designed using the company’s Probe Designer software on the canine galectin-3 transcript sequence (Ensembl assession number ENSCAFG00000015013), and labelled with the CAL Fluor Red 610 fluorophore. Labeling was performed on tissue specimens following the manufacturer’s instructions. Formalin-fixed and paraffin-embedded tissue samples were deparaffinised in xylene and rehydrated in a series of alcohols. This was followed by probe hybridization (1 μl from undiluted stock) overnight at 37°C. The following day, cells were washed with 2× saline sodium citrate (SSC), nuclei were counterstained with DAPI and cover glasses were mounted in Vectashield (Vector) on glass slides.

### Animal Tissues

#### Dogs

Primary and metastatic canine mammary tumors were obtained from female dogs submitted to surgery at the Small Animal Clinic of ICBAS-UP, UPVET and necropsies performed to the same animals whenever they were euthanized at owner’s request. Tumors were removed and fixed in 10% neutral buffered formalin. After being dehydrated and embedded in paraffin, a section of 4μm was obtained from each representative paraffin block for hematoxylin and eosin staining and immunohistochemistry procedures.

#### Experimental nude mice

Experimental nude mice, N: NIH(s) II-nu/nu—mice, were inoculated in the mammary fat pad with a suspension of 10^6^ cells of CMT-U27 cell line. Once the primary tumor xenograft attained the volume of 1 cm^3^, it was excised under general anesthesia. Mice were anesthetized by IP administration of 100 μL of a mixture containing 50 mg/kg of Ketamin (IMALGENE 1000) and 1 mg/kg of medetomidine hydrochloride (Medetor) and the tumor was excised. Atipamezole, 2.5 mg/kg (Revertor) was used *per* mice to antagonize the effect of anesthesia. Mice were subsequently treated with an oral solution of 10 mg/kg of tramadol chloridrate (Tramal) every 8h for 24–48h to prevent pain. The animals were humanely euthanized whenever their body weight started to decrease or any signs of poor body condition were shown. All studies with experimental animals were approved by the ethics committee of the University of Porto and were carried out in accordance with the European Guidelines for the Care and Use of Laboratory Animals, Directive 2010/63/UE and the National Regulation published in 2013 (Decreto-Lei n.° 113/2013 de 7 de Agosto).

### Immunohistochemistry

Galectin-3, GLUT-1, MUC1 and CD31 expression were evaluated in formalin-fixed, paraffin-embedded specimens of both spontaneously occurring CMT and CMT-U27 tumor xenografts using immunohistochemistry following a standard protocol. Briefly, slides were deparaffinized in xylene and rehydrated in an ethanol/water gradient. Antigen retrieval was performed with 0.005% of extran for 8 minutes in microwave boiling cycles and then slides were cooled off in PBS for five minutes. Endogenous peroxidase activity was blocked by 3% H_2_O_2_ in methanol solution for 10 minutes.

For GLUT-1 detection, sections were incubated for 2 hours at room temperature with a polyclonal anti-GLUT-1 (Abcam, diluted 1:400), washed and developed using the HRP polymer (Envision—Dako, Denmark) according to the manufacturer’ s instructions. To stain galectin-3, MUC1 and CD31, slides were incubated overnight at 4°C with a monoclonal antibody (M3/38 Bio science diluted 1:200, which only recognizes N-terminal domain of the protein), MUC1 (Santa Cruz Biotechnologies, diluted 1:50) and CD31 (Empresa, diluted 1:50). After incubation with the appropriate secondary antibodies for 30 minutes at room temperature, the Avidin-Biotin Complex (Vector Labs) amplification system was applied prior to detection. Slides were developed with DAB substrate (Sigma).

To identify hypoxic areas *in vivo*, we used the Hypoxyprobe-1 Kit (Chemicon International) diluted (1:50), as described by the manufacturer. Slides were lightly counterstained with hematoxylin, dehydrated, and mounted using histologic mounting media, Histomount (National Diagnostics). A negative control, without primary antibody, was included. All stained sections were examined under a light microscopy by three observers (de Oliveira JT.; Ribeiro, A.; and Gartner, F.).

### Double-labeling Immunofluorescence in tissue sections

For simultaneous visualization of galectin-3 with GLUT-1 on the same tissue section, a double-labeling immunofluorescence was performed. Necrotic tissue exhibiting sections were chosen by the pathologist (Gartner, F.). Briefly after blocking with normal serum in 10% BSA for 30 minutes, sections were incubated with the first primary antibodies rat anti-galectin-3 (Bioscience) 1:200 in 5% BSA and rabbit anti-GLUT-1 (Abcam) 1:100 overnight at 4°C, they were then washed in PBS and incubated with Alexa 488-conjugated with a donkey anti-rat antibody (1:200),(Invitrogen) and Alexa 594-conjugated with a goat anti-rabbit antibody (1:200),(Invitrogen) respectively for 45 min. All sections were then incubated with 1:100 PBS diluted 4’-6-Diamidino-2-phenylindole (DAPI) for 15 min. Finally slides were mounted in glycerol-based Vectashield medium (Vector, Burlingame, CA). Slides were analyzed with a Zeiss fluorescence microscope.

### Statistical Analysis

Whenever appropriate, the results are presented as mean ± standard deviation. Statistical analysis was done with Student’s unpaired t-test using GraphPad Prism 5.02 version. Differences were considered statistically significant at P<0.05.

## Results

### Galectin-3 is up-regulated in CMT-U27 cells exposed to hypoxic conditions

**Fig 1:**
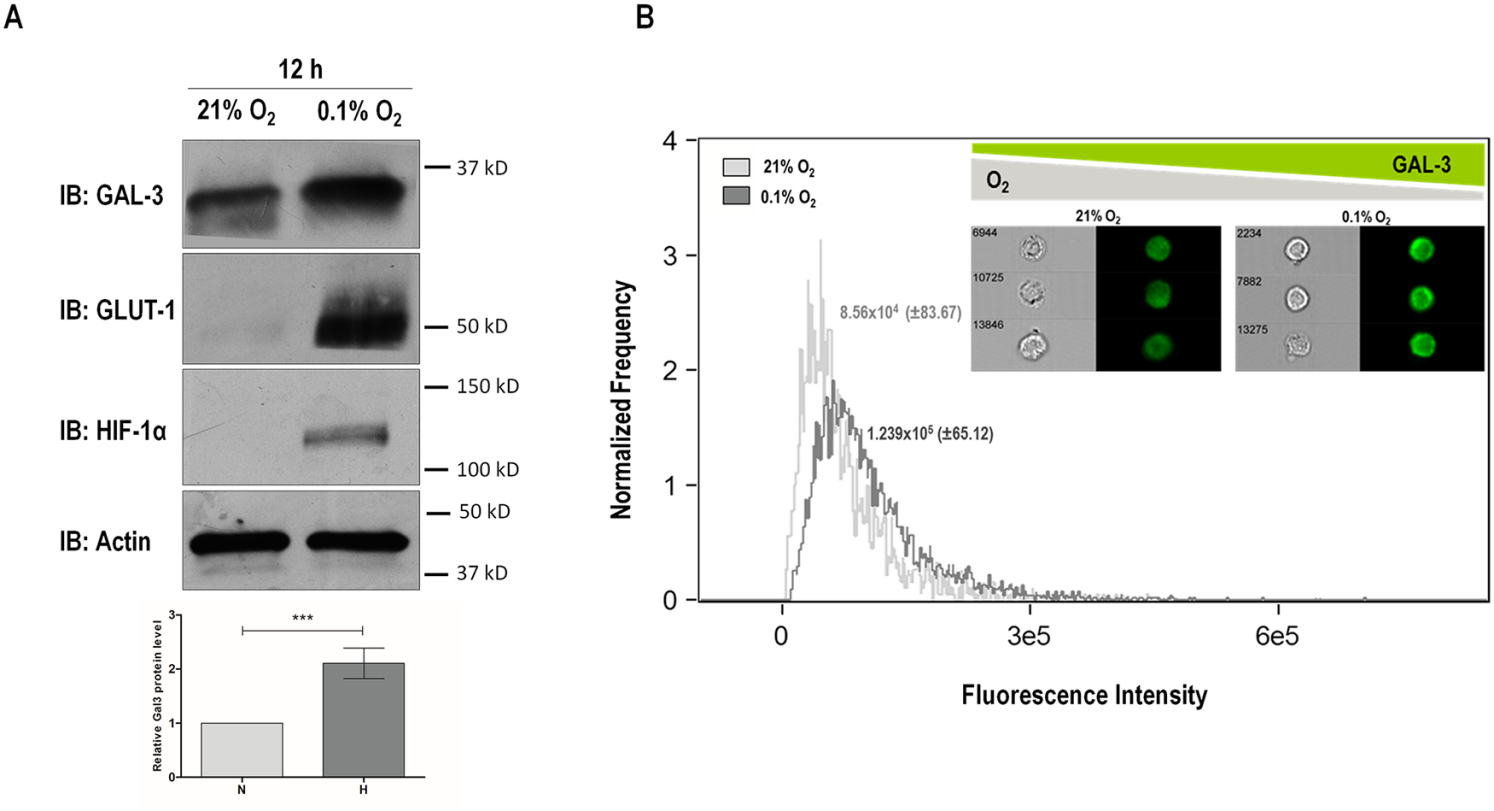
Galectin-3 expression in CMT-U27 cell line under hypoxic conditions. Galectin-3 protein expression was assessed by western blot and FACS using an anti-galectin-3 antibody (M3/38) **(A)** Western Blot analyses show increased expression levels of galectin-3 (p<0.001), HIF1-α and GLUT-1 in protein extracts from malignant CMT-U27 cell line after 6 hours 0.1% oxygen exposure. Relative intensity of the indicated protein level bands were normalized to actin. **(B)** FACS analysis of galectin-3 expression on CMT-U27 cells. CMT-U27 cells exposed to hypoxia and controls were stained with M3/38 anti-galectin-3 antibody. Galectin-3 expression was measured by florescence intensity of each cell, representative cells correspond statistically to median fluorescence of all cells. Galectin-3 was increased in CMT-U27 cells under hypoxia (1.238x10^5^± mean fluorescence intensity) when compared with cells kept in normoxia (8.56x10^4^± mean fluorescence intensity).

Up-regulation of galectin-3 under hypoxia has been described in a non-neoplastic context [20]. In order to assess a possible regulation of galectin-3 by hypoxia in neoplastic settings, we used a highly metastatic canine mammary cancer cell line, CMT-U27. CMT-U27 cells were cultured for 12 hours in hypoxia (0.1% O_2_) or normoxia (21% O_2_) as a control condition. HIF-1α and GLUT-1 protein expression were also determined by Western Blot as hypoxia readouts [34, 35]. Galectin-3 expression was significantly increased (p<0.001) in protein extracts from CMT-U27 cells exposed to hypoxia for 12 hours (paralleled by HIF-1α and GLUT-1 increased expression) when compared with normoxic controls (Fig 1A). In accordance, FACS analysis showed increased galectin-3 expression in CMT-U27 cells exposed to 0.1% oxygen (1.239x10^5^±83.67 mean fluorescence intensity) when compared with cells kept in normoxia (8.25x10^4^±65.12 mean fluorescence intensity) (Fig 1B). Galectin-3 expression was also increased in protein extracts of hypoxic CMT-U27 cells after 12 hours but not after 24 hours of 0.1% oxygen exposure (S1 Fig). Despite galectin-3 may be secreted to the extracellular space in an energy-independent manner [36], our present data seem not to support any additional differences in the secretion of the lectin at different points of hypoxia exposure (S2 Fig). Likewise, no differences were found in the expression of galectin-3 when CMT-U27 cells were treated with a proteasome inhibitor, either in normoxic or in hypoxic conditions (S3 Fig).

### Catalase treatment prevents CMT-U27 cells under hypoxia from up-regulating galectin-3

**Fig 2:**
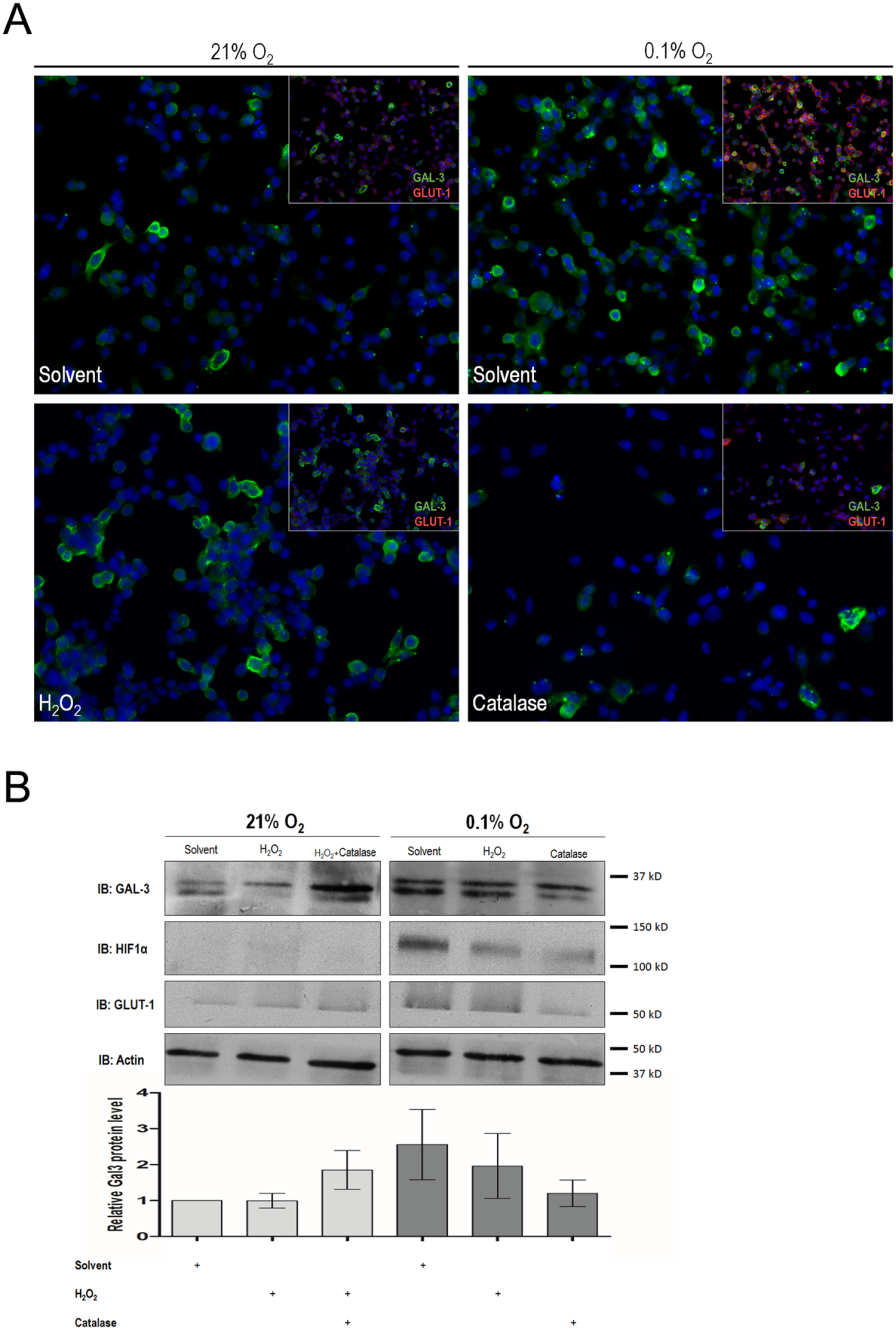
Galectin-3 expression under oxidative stress conditions in hypoxic and normoxic cells. **(A)** Galectin-3 subcellular expression was assessed by double-labelling immunofluorescence. Representative galectin-3 cell expression shows that despite galectin-3 increase under hypoxia, in the presence of catalase this was not verified. GLUT-1, included as a well-known target of hypoxic conditions, was also not increased in the presence of catalase. **(B)** To quantitatively evaluate galectin-3 expression under different oxidative stress conditions western blot analyses were performed. Relative intensity of the indicated protein level bands normalizes to actin were measured. In hypoxic conditions, in the presence of catalase, increased expression of galectin-3 was not observed in CMT-U27 cells. Galectin-3 expression, was kept, both in levels and quantity, similar to that seen in normoxic cells. However, in normoxia, catalase itself appears to increase the expression of galectin-3. In normoxic conditions, hydrogen peroxide treatment induced an apparent increase of a heavier molecular weight form of galectin-3 but had no additive effect on the increase of galectin-3 under hypoxia.

One of the cellular outcomes of hypoxic conditions is oxidative stress, namely the increase in reactive oxygen species (ROS) production. We hypothesized that the increase in galectin-3 expression in hypoxia could, at least to some extent be, due to oxidative stress in CMT. To test this, cells were submitted to hydrogen peroxide and/or catalase treatment, to induce oxidative stress and to detoxify, respectively. Double-labeling immunofluorescence analyses were performed to evaluate simultaneous expression of galectin-3 and GLUT-1 in normoxic and hypoxic catalase-treated CMT-U27 cells. Galectin-3 and GLUT-1 presence, as evidenced by fluorescent immunostaining, was simultaneously increased in hypoxic CMT-U27 cells when compared with normoxic controls. However, catalase-treated CMT-U27 hypoxic cells failed to show such an increase of galectin-3 and also showed a lower of an increase of GLUT-1 immunostaining (Fig 2A). Galectin-3 expression was analyzed in the CMT-U27 cell line treated with hydrogen peroxide, using Western Blot analysis (Fig 2B). An increased heavier form of galectin-3 was observed in hydrogen peroxide-treated cells kept under normoxic conditions. Next, to detoxify the culture medium, catalase was used to counteract the effect of endogenous H_2_O_2_, under otherwise hypoxic and normoxic conditions. Upon 12 hours treatment with 4μM of catalase, galectin-3 expression was overall increased in protein extracts of CMT-U27 cells under normoxia. In hypoxic conditions, upon medium detoxification, galectin-3 expression decreased when compared with hypoxic untreated CMT-U27 cells (Fig 2B), corroborating the immunofluorescence analyses.

### Cytoplasmic galectin-3 localization is increased under hypoxic conditions

**Fig 3:**
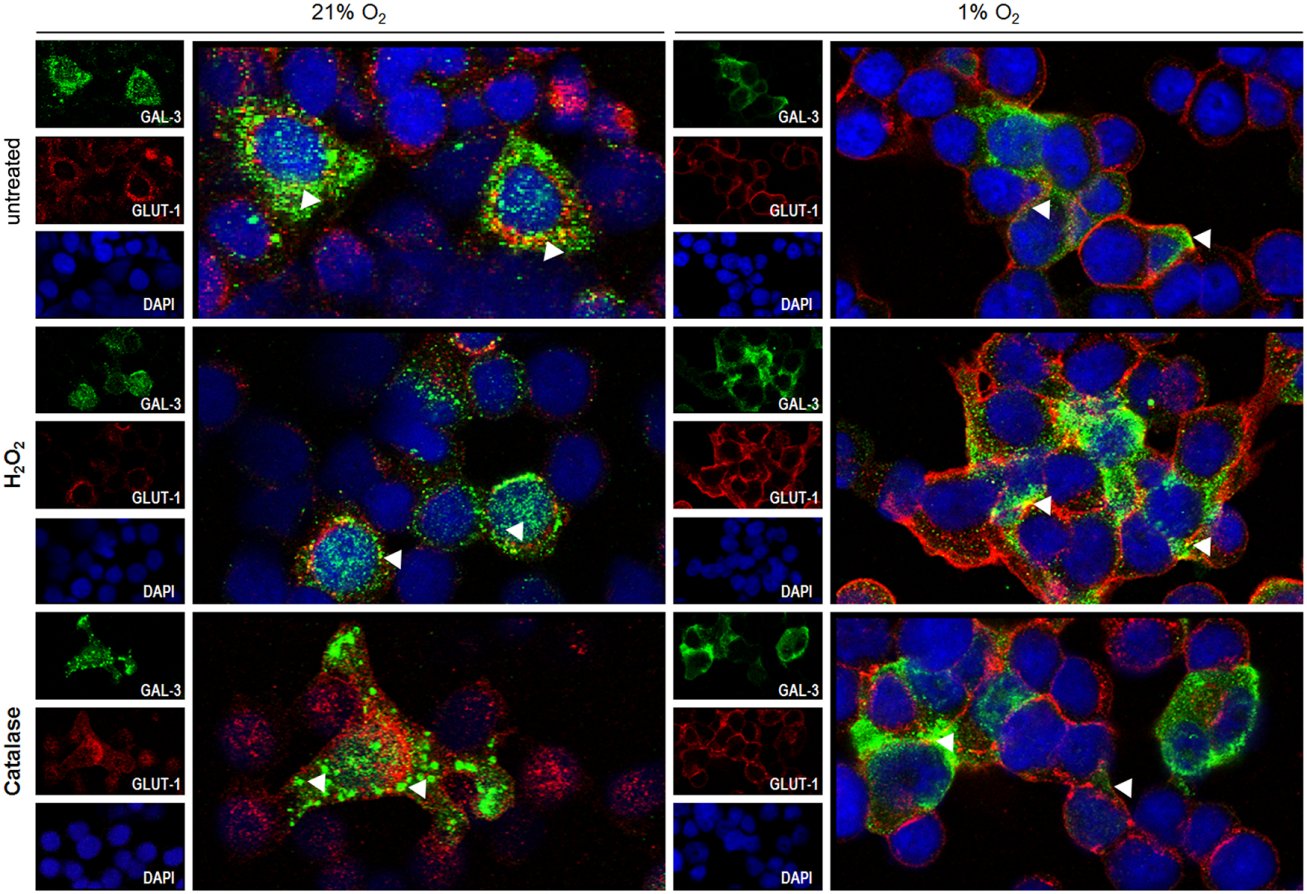
Subcellular localization of galectin-3 and GLUT-1. Subcellular localization of galectin-3 (green color) and GLUT-1 (red color) under normoxic and hypoxic conditions was assessed by double-labeling immunofluorescence and observed by laser scanning confocal microscopy. Under normoxic conditions galectin-3 had a nuclear and cytoplasmic localization. However, in hypoxic conditions galectin-3 expression was mainly cytoplasmic. Galectin-3 localization differed in hydrogen peroxide and catalase treated cells under normoxic and hypoxic conditions. Following hydrogen peroxide treatment in CMT-U27 cells under normal oxygen conditions, galectin-3 left the cytoplasm and was mainly localized in the nucleus. Nevertheless, in hydrogen peroxide treated cells under hypoxia galectin-3 was mostly cytoplasmic. Catalase treated CMT-U27 cells under normoxia presented galectin-3 in specific organelles; however in catalase-treated hypoxic cells galectin-3 localization was again mostly cytoplasmic.

Galectin-3 actions rely on its subcellular location and on the phenotype of the cell itself, ranging from a pro-apoptotic function effect when present in the nucleus, to an anti-apoptotic one when in the cytoplasm [37]. As a cell membrane protein, it also regulates cell-cell and/or cell-extracellular matrix adhesion [38]. To assess a putative effect of hypoxia and oxidative stress on the subcellular location of galectin-3, we analyzed galectin-3 and GLUT-1 double-labeled cells under a confocal microscope (Fig 3). Untreated normoxic cells expressed galectin-3 in the cytoplasm and to a lesser extent in the nucleus. However when untreated CMT-U27 cells were exposed to hypoxia galectin-3 nuclear expression could no longer be found. Cells treated with H_2_O_2_, under normal oxygen conditions, showed increased nuclear galectin-3 expression. However, when cells were exposed to both H_2_O_2_ and hypoxia, nuclear galectin-3 expression was lost, its subcellular localization being again mainly cytoplasmic. Under these conditions, in a few CMT-U27 cells, particularly in focal contact points, galectin-3 was co-located with GLUT-1 at the cell membrane. Finally, catalase-treated CMT-U27 cells under normal oxygen conditions expressed galectin-3 in specific organelles of irregularly-shaped cells, while cells under hypoxic conditions (1% O_2_) cells retained their morphology and, when present, a cytoplasmic location of galectin-3 was found (Fig 3).

### Galectin-3 transcription is increased in CMT-U27 cells under hypoxia

**Fig 4:**
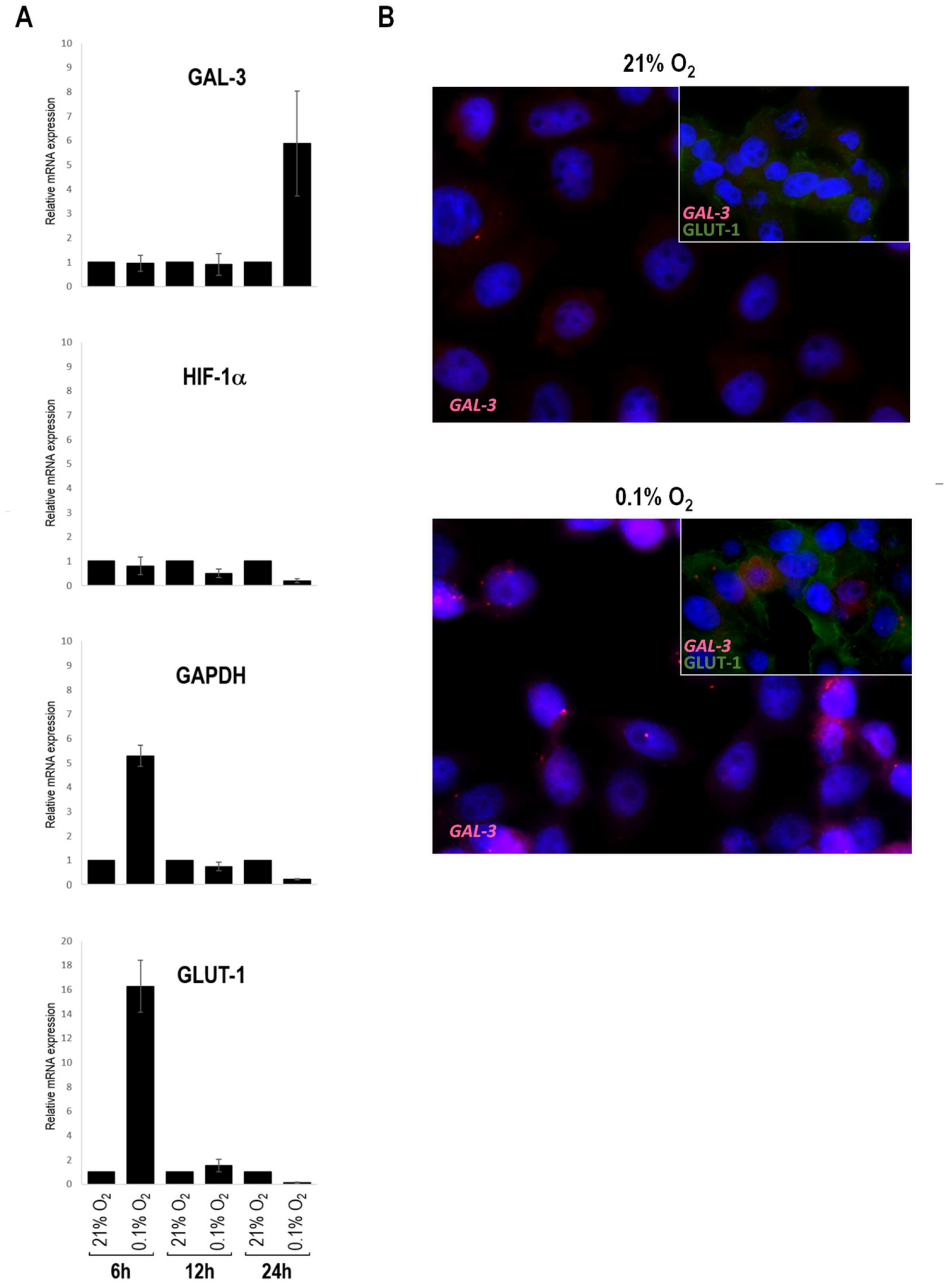
Galectin-3 mRNA expression under hypoxic conditions. **(A) m**RNA extracted from malignant CMT-U27 cell line was converted to cDNA and analyzed by real-time polymerase chain reaction (PCR) to assess quantitatively galectin-3 expression in CMT-U27 cells under hypoxic conditions. An increase in galectin-3 mRNA was observed only upon 24 hours of hypoxia treatment. No differences were seen in the transcription of HIF-1α, however the transcription of GAPDH and GLUT-1 seemed to early respond to hypoxia. cDNA contents were normalized on the basis of predetermined levels of 18S. **(B)** Representative mRNA expression of galectin-3, was visualized using a set of Stellaris RNA fluorescence *in situ* hybridization probes. GLUT-1 (green color) was used as a hypoxia control. Blue color shows the nucleus stained by DAPI. CMT-U27 cells exposed for 24 hours to hypoxia presented intense red FISH signals that are predominantly located in the cytoplasm and reflect the galectin-3 mRNA. Each spot corresponds to a single mRNA molecule.

In order to determine whether the differing levels of galectin-3 expression levels reflected transcriptional regulation, we measured the lectin’s mRNA levels in CMT-U27 cells upon 6, 12, and 24 hours of hypoxia exposure. In fact, cells displayed higher expression levels of galectin-3 mRNA (4-fold increase) upon 24 hours of hypoxia exposure when compared with normoxic controls. We further investigated HIF-1α, GLUT-1 and GAPDH transcription in the hypoxic cells. After 6 hours of hypoxia, GLUT-1 and GAPDH mRNA levels were found to be 16- and 4-fold increased, respectively. When compared with normoxic cells, HIF-1α mRNA levels were not significantly altered at the studied time points (Fig 4A). To further assess galectin-3 mRNA expression by cells under hypoxic conditions (0.1% O_2_ for 24 hours), FISH analyses were performed, showing an increase of galectin-3 mRNA expression in a subset of the cells (Fig 4B). These results suggest that changes in of galectin-3 transcription may be a late response to hypoxia, when compared with GLUT-1 and GAPDH, in malignant CMT.

### Hypoxia-induced expression of galectin-3 in mammary tumor xenografts

**Fig 5:**
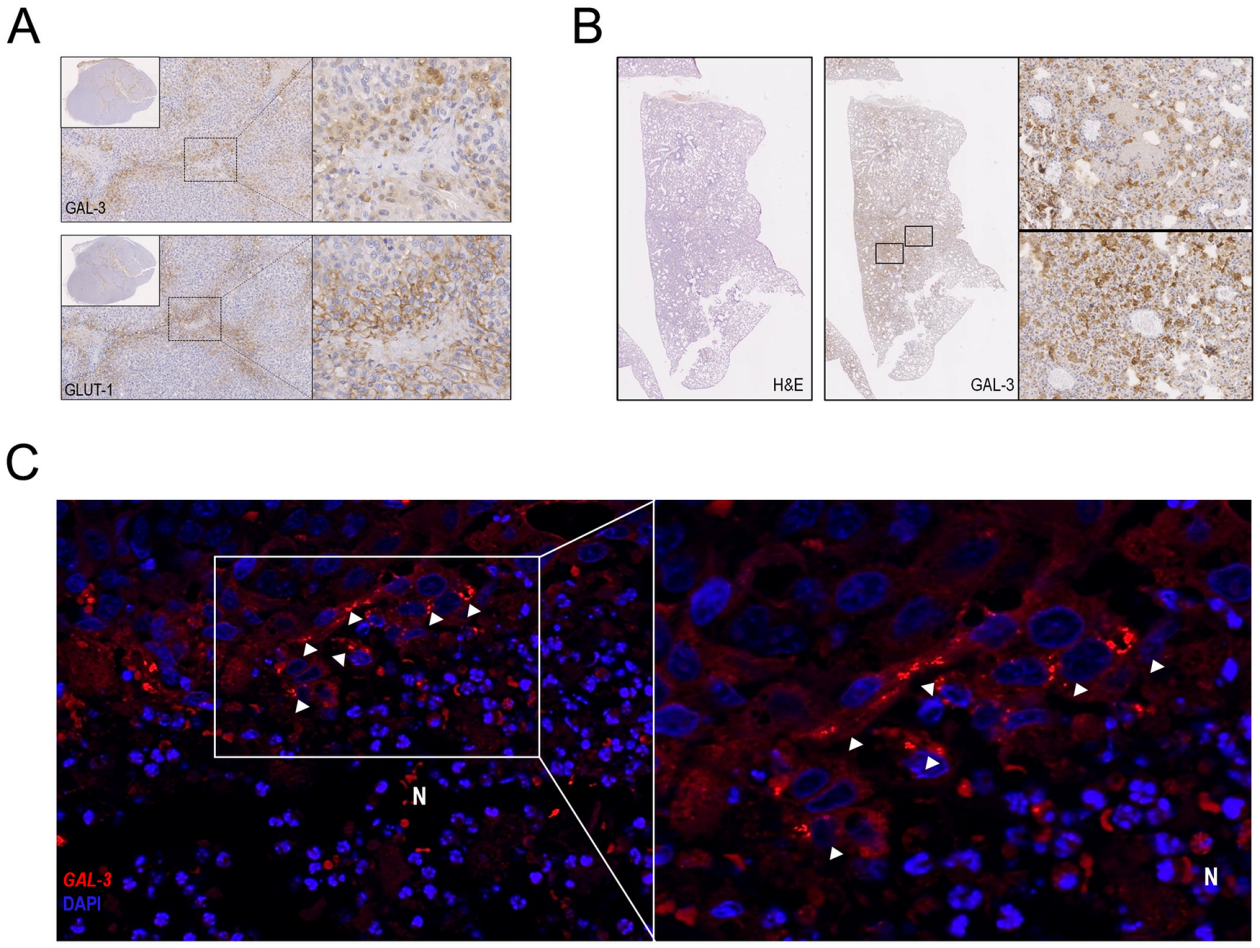
Protein and mRNA galectin-3 expression are up regulated in tumor cells surrounding necrotic areas of CMT-U27 mice xenografts. Female N: NIHY(s)II-nu/nu mice were inoculated subcutaneously in the mammary fat pad with a suspension of 10^6^ cells of the malignant CMT-U27 cell line. Primary tumors and metastases were collected at different times after inoculation. (A) Immunohistochemistry was performed to study galectin-3 and GLUT-1 expression (brown color) using M3/38 anti-galectin-3 and anti-GLUT-1 antibodies. Galectin-3 and GLUT-1 were overexpressed in viable tumor cells surrounding necrosis. **(B)** Galectin-3 was also overexpressed in lung micro metastases **(C)** Galectin-3 mRNA expression was detected *in situ* using a set of Stellaris RNA fluorescence in situ hybridization (FISH) probes in the cytoplasm of cells surrounding necrotic areas in CMT-U27 primary tumor xenografts. Nucleus were stained by DAPI. Each red spot corresponds to a single galectin-3 mRNA molecule.

Nude mice solid tumor xenografts often display necrosis, presumably due to the existence of highly hypoxic areas [39]. To evaluate the existence of such hypoxic areas and whether this would also influence galectin-3 expression *in vivo*, we inoculated the CMT-U27 cell line into the mammary fat pad of female nude mice and allowed the tumors to grow until they reached approximately 1000 mm^3^. Galectin-3 and GLUT-1 (hypoxia marker) expressions were assessed by immunohistochemistry in the tumor xenografts. Large necrotic areas were found and appear to be oxygen deprived as evidenced by GLUT-1 overexpression. High levels of galectin-3 expression were found in tumor areas surrounding necrotic tissue (Fig 5A) as well as in lung micro metastases (Fig 5B). To determine the presence and abundance of galectin-3 mRNA in CMT-U27 xenograft tumor cells surrounding necrosis, FISH was performed. Galectin-3 mRNA was found to be increased in cells surrounding necrotic areas (Fig 5C).

### Co-expression of galectin-3 and GLUT-1 in cell subpopulations of highly hypoxic areas in primary tumors and well-established metastases

**Fig 6:**
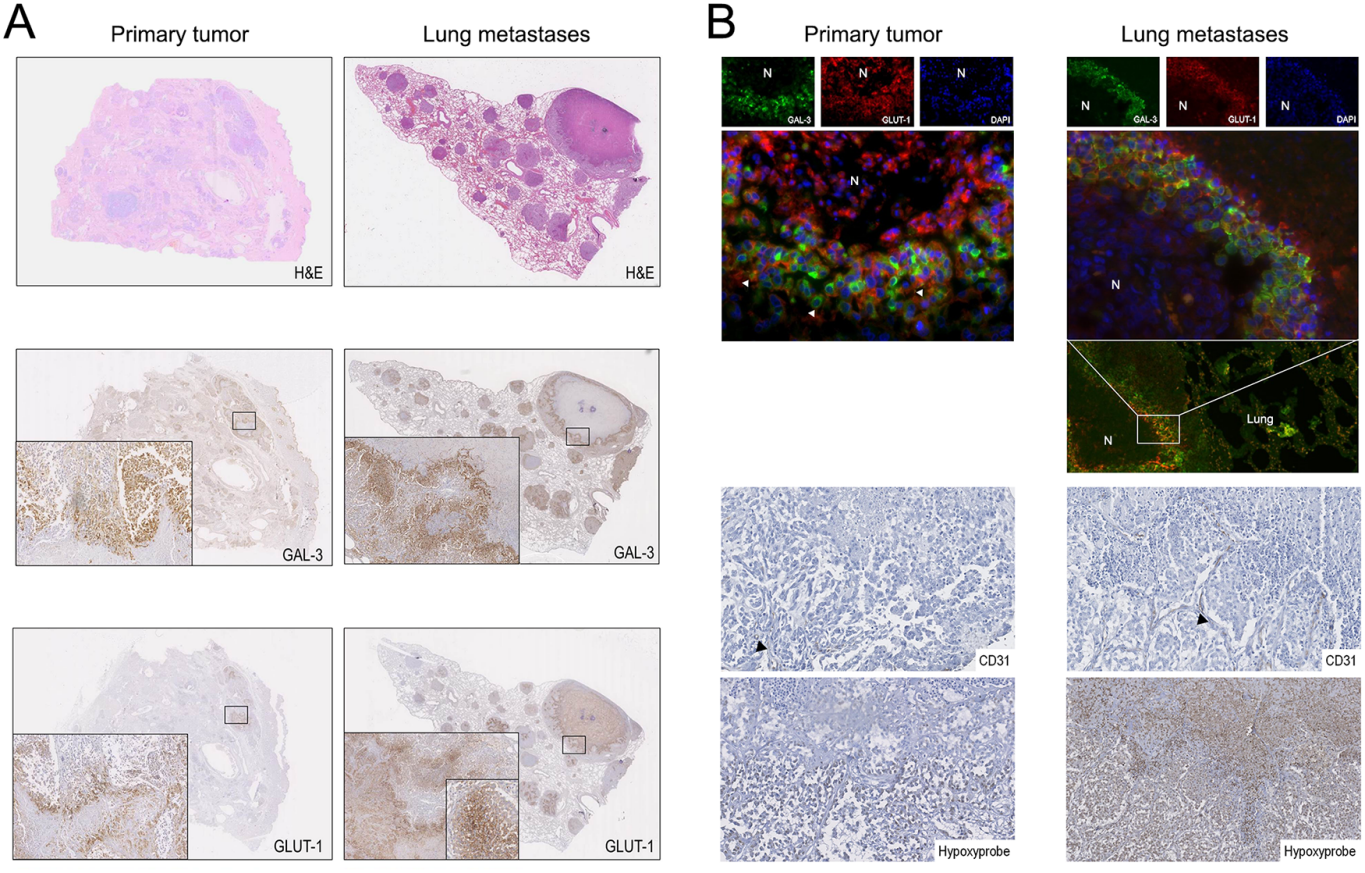
Galectin-3 and GLUT-1 are up regulated in hypoxic tumor cells surrounding necrotic areas both in primary malignant CMT and lung metastases. **(A)** Photomicrographs depict galectin-3 and GLUT-1 immunostaining (brown color) with hematoxilin counterstain in malignant CMT and lung metastasis. Galectin-3 and GLUT-1 were expressed in viable tumor cells surrounding necrosis (N). **(B)** Co-expression between galectin-3 (green color) and GLUT-1 (red color) was assessed by double-labeling immunofluorescence. Co-expression (yellow color) was observed both in viable tumor cells surrounding necrotic areas (N) of primary tumors and lung metastases. Pimonidazole hydrochloride (Hypoxyprobe) was used as a hypoxia marker. High hypoxic staining and virtually no CD31 staining, showing endotelial cells, further confirmed low oxygen tension in necrosis-surrounding areas.

Tumor xenograft models present an overly rapid volume doubling time, often failing to model key steps of the metastatic process. Galectin-3 expression in areas surrounding necrosis was proposed to be associated to invasiveness [12, 31]. In order to investigate if oxygen deprivation would be a steering factor underlying galectin-3 expression and function in spontaneously occurring mammary tumors, a small series of metastatic CMT was examined. Fig 6 shows necrotic areas in primary CMT cases and metastatic lesions. Galectin-3 and GLUT-1 were both overexpressed in viable tumor cells surrounding such areas (Fig 6A).

Galectin-3 and GLUT-1 were co-expressed in hypoxic cells both in primary tumors and well-established lung metastases (Fig 6B). Hypoxyprobe-1 was included to confirm that this tissue was low in O_2_ tension and CD31 staining further showed lack of significant neovascularization in the necrosis-surrounding areas (Fig 6B). These results in a spontaneous model further point to an important hypoxia regulation of galectin-3, possibly associated to cancer aggressiveness.

## Discussion

In this study, we used the spontaneous malignant CMT to investigate a possible role of hypoxia in regulating galectin-3 during the metastatic process and demonstrated that: (1) galectin-3 expression is up-regulated in malignant CMT cells under hypoxia; (2) catalase treatment almost completely precludes hypoxia-dependent up-regulation of galectin-3; (3) galectin-3 nuclear location is lost under hypoxic conditions; (4) galectin-3 increased mRNA transcription is a late response to hypoxia and is associated to specific subpopulations of malignant CMT cells; (5) galectin-3 is overexpressed *in vivo* in hypoxic necrosis-surrounding areas and down-regulated in most other areas of the primary tumor or well-established metastases, with the interesting exception of micro metastases which overexpress galectin-3.

Our data showed an increase in galectin-3 protein expression in CMT-U27 cells exposed to hypoxia. Several studies demonstrated the influence of hypoxia in the up regulation of galectin-3 in non-neoplastic cells such as the nucleus pulposus of the intervertebral disc [20], the human placental cell line BeWo under hypoxia [40] and an ischemic model of perinatal brain injury [41]. Both the existence of a HIF-1α binding site in the galectin-3 promoter region [20] and the galectin-3 up-regulation in mice fibroblasts transfected with HIF [18] point to a crucial role of the molecule in galectin-3 regulation. To the best of our knowledge, this is the first work showing a galectin-3 up-regulation in hypoxic cancer cells, here in a mammary model.

When submitted to hypoxia in the presence of catalase, CMT-U27 cells expressed galectin-3 similar to normoxic untreated cells. Previous studies yielded contradictory results. Catalase overexpression had no clear effects on the expression or transcriptional activity of HIF1 in human hepatoma cells [42]. Nevertheless, the opposite has been shown in human lung epithelial A549 cells [43]. Qutub and Popel suggested, when using an experimentally-based computational model of HIF1α degradation, that increased intracellular catalase may not have a pronounced effect on the response to hypoxia via direct HIF1 activation, while extracellularly added H_2_O_2_ and catalase levels most likely do [44].

Given that catalase induces apoptosis in MCF7 breast cancer cells [45] and that galectin-3 has an anti apoptotic role [46] it is possible that that the up-regulation of galectin-3 is a cell survival mechanism against the catalase-induced apoptosis. Further supporting such hypothesis, under conditions of normoxia treatment with catalase not only galectin-3 protein levels increased but also led to an accumulation of galectin-3 in specific organelles. After apoptotic stimuli, galectin-3 locates preferably in the perinuclear mitochondrial membranes, a phenomenon mediated by synexin, where it regulates mitochondrial integrity by preventing its damage and cytochrome c release, thus avoiding apoptosis [47]. Moreover, an adenovirus containing catalase was shown to affect the activity of apoptosis signal-regulating kinase 1, which activity is dependent on H_2_O_2_ and indirectly related to HIF1 in pancreatic cancer cells [48].

This hint that detoxification of the medium could have been important for the regulation of galectin-3 led us to examine the role of ROS, in particular hydrogen peroxide, under hypoxic conditions. Although in normoxic conditions hydrogen peroxide induced an apparent increase of a heavier molecular weight form of galectin-3, such effect was not observed under hypoxia. Galectin-3 phosphorylation is involved in its nuclear transport [6]. Galectin-3 localization was deemed to have different effects: in the nucleus it has been described to play a pro-apoptotic role while cytoplasmic galectin-3 inhibits apoptosis [38, 46, 49, 50] and when in the inner cytoplasmic membrane it binds and maintains the oncogenic RAS activity involved in actin cytoskeletal integrity, cell adhesion, apoptosis and cell migration among others [51]. Altogether, these results suggest that the shift towards a cytoplasmic and membranous localization of galectin-3 favors resistance to apoptosis and malignancy of CMT-U27 tumor cells under hypoxia.

We observed that galectin-3 mRNA was increased upon low oxygen exposure, demonstrating that the gene was transcriptionally activated under hypoxic conditions. Our studies are in agreement with an earlier finding of an increase in galectin-3 mRNA expression in HeLa cells exposed to hypoxia and ischemia [41]. However, although protein expression of galectin-3 was increased as early as after 6 hours of hypoxia, such was not evident at the mRNA level before 24 hours of treatment, pointing out a complex expression regulation, both at transcriptional and post-transcriptional levels. For the latter, we envisaged at least three possible hypotheses to justify the early increase in protein expression: a) differences in secretion, following which galectin-3 would be less secreted in hypoxia than in normoxia; b) a lessened galectin-3 protein degradation by the proteasome under hypoxia; c) differential activity and / or expression of micro RNAs under hypoxic conditions would influence the translation of galectin-3 from its messenger RNA. However, as the gene is also transcriptionally regulated, though at a later stage, it probably ensures a more sustained and long-lasting expression of the protein under such conditions, possibly impacting on tumor aggressiveness. Our current data do not seem to support the first two hypotheses. Regarding the third hypothesis, galectin-3 is regulated by MUC1 through microRNA in human cells, in a glycosylation-dependent manner [52]. *In situ* hybridization has allowed us to determine the existence of a heterogeneous population of malignant CMT-U27 cells in what concerns the expression of galectin-3 mRNA under hypoxic conditions, with subpopulations often exhibiting different glycoconjugate profiles. Further studies are thus warranted to ascertain whether MUC1-dependent galectin-3 regulation, previously described by our group in CMT [7], is also dependent on a micro-RNA and whether this is related to hypoxia. This would be feasible since MUC1 itself is overexpressed under hypoxia (S4 Fig) [53, 54].

The mRNA analysis following hypoxia showed no differences in the transcription of HIF1-α, itself. Under normoxic conditions, HIF-1α is hydroxylated leading to its proteasome degradation [18, 25, 42] but hypoxia inhibits the degradation process. HIF-1α is then transported to the nucleus, where it binds to hypoxia-responsive element sequences (HRE) in the promoter region of target genes, mainly involved in adaptive changes that allow tumor cells to survive and proliferate in a hypoxic environment, thus contributing to the malignant phenotype and to aggressive tumor behavior [55, 56]. Thus, our results are in agreement with previous reports in the literature demonstrating that the regulation of this protein occurs by degradation/non degradation mediated by ubiquitination rather than by transcription [57], as it is constitutively expressed in the cells. On the opposite hand, the transcription of both GLUT-1 and GAPDH was highly increased following a short period of hypoxia (6 hours), suggesting they play a role as early responders to this type of stress, probably then perpetuating their effect over time. This early response could further be explained by the previous demonstration that GLUT-1 is a direct transcriptional target of HIF1, which rapidly increases upon any hypoxic pulse. The later transcriptional activation of galectin-3 could by justified by the need of co-factors up regulated later in the cascade of events following oxygen deprivation, although further studies are needed to prove it.

Previously, our group had described increased galectin-3 expression in viable cells of mammary carcinomas surrounding necrotic areas [12]. These results found support by findings by other groups in intraductal breast cancer which suggested that such increase was due to hypoxia, without however having demonstrated oxygen deprivation [31]. In this study we showed that the markedly necrotic areas around which galectin-3 is overexpressed in tumor cells as confirmed, as confirmed by the hypoxiprobe-1 staining in the same areas. Necrotic areas are often invaded by polymorphonuclear infiltrates [58]. Since leucocytes produce and secrete galectin-3, we thought it to be crucial to confirm whether the increased expression of galectin-3 in these regions would come from a tumor cell uptake of galectin-3 from the medium [59], or whether it was actually due to galectin-3 production by the tumor cells themselves. *In situ* hybridization confirmed that galectin-3 transcription was in fact increased in the tumor cells from hypoxic regions. Relevantly supporting the galectin-3 regulation by hypoxia, almost all tumor areas away from necrotic tissues in mice were consistently negative for galectin-3 and GLUT-1 labeling in all primary lesions. Interesting exceptions were lung micro metastatic colonies of CMT-U27 cells in which galectin-3 was almost always expressed. Our previous work regarding the differential expression of galectins-1 and -3 during the metastatic process in CMT pointed to the existence of hybrid but mainly galectin-3-expressing tumor cells in circulation which, after an initial homing period, gradually lose galectin-3 expression and gain that of galectin-1 [60]. Tumor xenografts grow rapidly and thus hypoxia sets in early and remains constant in the microenvironment. This is due to a high rate of tumor cell proliferation [39] that hampers the appropriate development of new blood vessels, essential to oxygenate and nourish tumor cells. Consequently, wide areas of necrosis emerge, with very low oxygen tensions and large amounts of metabolic wastes [18]. This prompted us to study spontaneous tumors as well, wherein tumor development is slower, potentially allowing more suitable angiogenesis to occur. Expression of galectin-3 was correlated with the expression of HIF-1α and VEGF in the development of hemangioblastoma [61]. In our series, spontaneous mammary carcinomas have shown some areas of hypoxia in specific locations suggesting that they may suffer different pressures of oxygen (intermittent hypoxia). This type of hypoxia has been described as inducing a more aggressive phenotype, with greater invasive ability, as cells have to constantly adapt to consecutive changes of the tumor microenvironment [62, 63]. Galectin-3 plays an important role in invasion, modulating the adhesion between cancer cells and ECM, as well as potentiating angiogenesis [1, 5, 22, 64].

There is a dynamic regulation of galectin-3 and its ligands in response to the tumor microenvironment [65]. The present study points to an important role of hypoxia in the later. It is now understood that anti-angiogenic therapy induces hypoxia and that hypoxia-dependent pathways lead to decreased cell death [66]. Galectin-3 might be an interesting target to overcome this [67].

## Supporting Information

S1 Fig Galectin-3 expression in hypoxic environment. Western Blot analyses show changes in the expression levels of galectin-3 at 12 hours but not at 24 hours. Proteins were extracted from the CMT-U27 cell line after exposed to hypoxia for 6, 12 and 24 hours. Relative intensity of the indicated protein level bands normalizes to actin were measured.
(TIFF)

S2 Fig Secreted galectin-3 expression. To confirm if galectin-3 was secreted to the extracellular space upon 24 hours of hypoxia, galectin-3 levels in the medium were evaluated by western blot. Galectin-3 was secreted into the extracellular space both in normoxia and hypoxia. No evidence of galectin-3 cleavage was found. Upon 24 hours of hypoxia exposure, galectin-3 was secreted to the medium.
(TIF)

S3 Fig Galectin-3 expression upon proteasome inhibition. Proteins were extracted from the CMT-U27 cell line after treatment with 25 μM MG132 (Calbiochem) or DMSO for 12 hours. No differences were observed in galectin-3 expression when cells were treated with a proteasome inhibitor.
(TIF)

S4 Fig Galectin-3 and the mammary tumor cell marker, MUC1 expression in lung metastasis. Photomicrographs depict Galectin-3 and MUC1 immunostaining in lung metastasis. MUC1 is a well-accepted marker of mammary tumor cells. Galectin-3 and MUC1 were expressed in tumor cells around necrosis.
(TIFF)
