# Supplementary figures and images for: Hypoxia Up-Regulates Galectin-3 in Mammary Tumor Progression and Metastasis
Source: PLoS One. 2015 Jul 29;10(7):e0134458. doi: 10.1371/journal.pone.0134458 (PMC4519331; doi:10.1371/journal.pone.0134458)

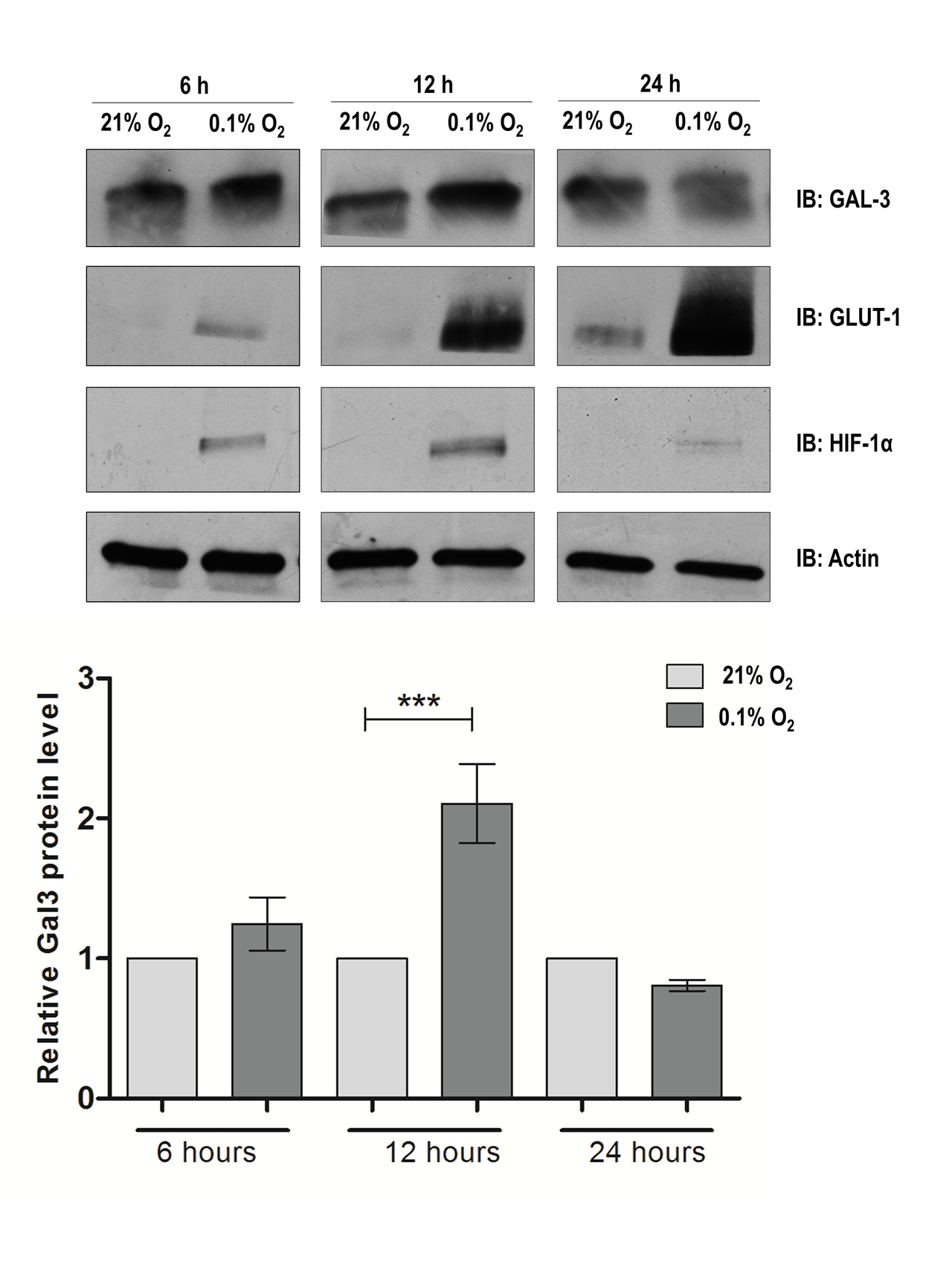

Supplement: S1 Fig — Western Blot analyses show changes in the expression levels of galectin-3 at 12 hours but not at 24 hours. Proteins were extracted from the CMT-U27 cell line after exposed to hypoxia for 6, 12 and 24 hours. Relative intensity of the indicated protein level bands normalizes to actin were measured. (TIFF) [file pone.0134458.s001.tiff]

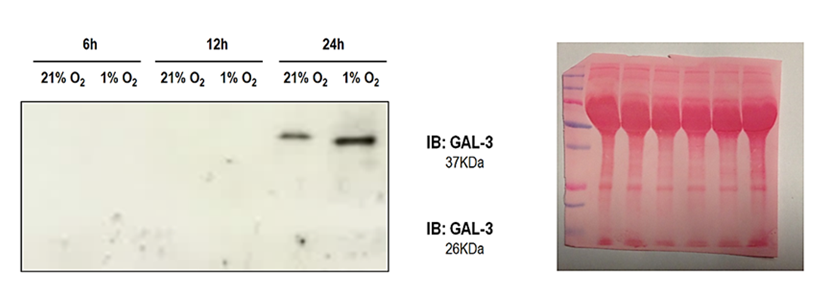

Supplement: S2 Fig — To confirm if galectin-3 was secreted to the extracellular space upon 24 hours of hypoxia, galectin-3 levels in the medium were evaluated by western blot. Galectin-3 was secreted into the extracellular space both in normoxia and hypoxia. No evidence of galectin-3 cleavage was found. Upon 24 hours of hypoxia exposure, galectin-3 was secreted to the medium. (TIF) [file pone.0134458.s002.tif]

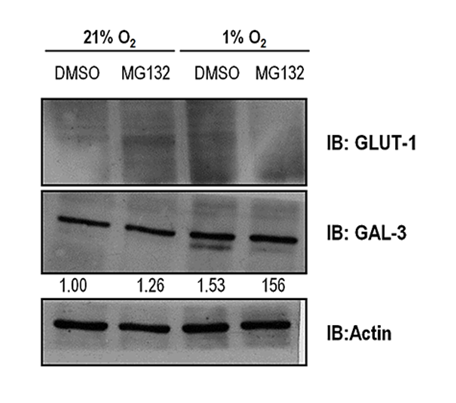

Supplement: S3 Fig — Proteins were extracted from the CMT-U27 cell line after treatment with 25 μM MG132 (Calbiochem) or DMSO for 12 hours. No differences were observed in galectin-3 expression when cells were treated with a proteasome inhibitor. (TIF) [file pone.0134458.s003.tif]

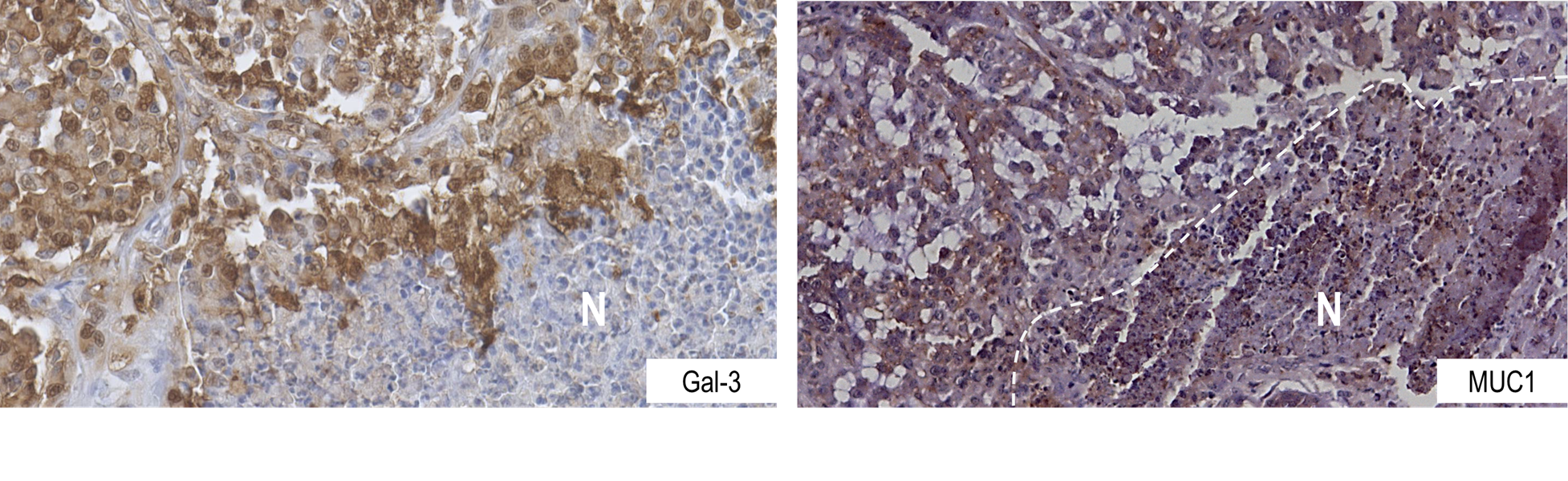

Supplement: S4 Fig — Photomicrographs depict Galectin-3 and MUC1 immunostaining in lung metastasis. MUC1 is a well-accepted marker of mammary tumor cells. Galectin-3 and MUC1 were expressed in tumor cells around necrosis. (TIFF) [file pone.0134458.s004.tiff]
